# Exploring the Impact of After-Hours Work Connectivity on Employee Performance: Insights from a Job Crafting Perspective

**DOI:** 10.3390/bs14111078

**Published:** 2024-11-11

**Authors:** Chuanhao Fan, Tianfeng Dong, Jiaxin Wang

**Affiliations:** Business School, Hohai University, Nanjing 211100, China; 221313040009@hhu.edu.cn (T.D.); 211313040044@hhu.edu.cn (J.W.)

**Keywords:** after-hours work connectivity, approach-oriented job crafting, avoidance-oriented job crafting, job performance, psychological contract

## Abstract

With the leapfrog development of information and communication technology and the intensification of external competition among enterprises, after-hours work connectivity through communication devices has become a new norm in the workplace. While it offers certain conveniences, the constant connectivity it entails also imposes significant pressure on employees. How to comprehensively understand and rationally treat after-hours work connectivity has become an issue that organizations need to pay great attention to. Based on conservation of resources theory, this study analyzed 407 questionnaires to explore the “double-edged sword” effect of after-hours work connectivity on employee performance and analyzed the moderating effect of the psychological contract. The results indicate the following: (1) Proactive pathway: after-hours work connectivity promotes employees’ job crafting behaviors toward approach-oriented adjustments, thereby enhancing job performance. (2) Passive pathway: after-hours work connectivity encourages employees’ job crafting behaviors toward avoidance-oriented adjustments, leading to decreased job performance. (3) The psychological contract positively moderates the relationship between after-hours work connectivity and approach-oriented job crafting and negatively moderates the relationship between after-hours work connectivity and avoidance-oriented job crafting, regulating both the positive and negative coping pathways. The research findings contribute to assisting organizations in adopting a dialectical perspective towards and effectively utilizing after-hours work connectivity. This aids in achieving a balance between organizational effectiveness and employee well-being, seeking a mutually beneficial work paradigm, and providing managerial recommendations to promote sustainable organizational development.

## 1. Introduction

With the rapid advancement of information and communication technology and the continuous iteration of communication devices, the convenience of remote communication methods such as email and instant messaging has been increasingly enhanced [1]. The widespread adoption of information and communication technology has shattered temporal and spatial barriers in information transmission among colleagues, leading to a profound transformation in the modes of work interaction. Even outside the conventional office setting, employees can still maintain contact with work using communication tools during non-work time, a phenomenon referred to as after-hours work connectivity [2,3]. After-hours work connectivity is gradually permeating into employees’ work and personal lives, providing convenience for their work while making it increasingly difficult to separate work from life. Particularly in the current macro trend of intensified external competition and economic downturn, the performance pressure on businesses and the anxiety of managers are transmitted to employees. This results in an increase in non-standard working hours for employees, who deal with work-related information even during rest and off-hours [4]. According to the “Work Overtime Status Survey Report 2022” released by 51job, 84.7% of professionals continue to engage with work-related information even after they have finished work. Deloitte’s “Global Human Capital Trends” research report further emphasizes that an era of constant work connectivity throughout the day has quietly arrived. “Resting without stopping” and “daring not to go offline” have become the new norms in the workplace, where utilizing information and communication technology for after-hours work connectivity is prevalent [5,6].

Through a literature review, previous research has predominantly examined the positive or negative impact mechanisms of after-hours work connectivity from singular perspectives. On the positive front, after-hours work connectivity behavior is perceived as a work resource. The behavior allows employees to complete urgent tasks anytime and anywhere, flexibly managing work content and schedule [7,8], thus enhancing their autonomy and sense of control [8,9]. This contributes to fostering workplace thriving and work–family synergy [10,11]. Conversely, on the negative side, after-hours work connectivity is regarded as an irresistible work demand. Increased “continuous connectivity” leads to longer working hours, increased workloads, and problems with boundaries between work and personal life. This is a serious drain on individual resources and leads to increased work pressure for employees. Employees transition from being “on-call at all times” to feeling “overwhelmed by demands,” eliciting negative emotional responses [12,13], impairing employees’ physical and mental well-being [14,15], precipitating work–family conflict [16], and negatively impacting employees’ work performance [17,18]. This manifests as reduced work engagement; eliciting feelings of work fatigue and withdrawal; as well as fostering counterproductive behaviors, time encroachment, procrastination, and absenteeism [17,19,20,21,22]. Therefore, after-hours work connectivity is a double-edged sword. Currently, after-hours work connectivity has become a prevalent workplace habit. Faced with its frequent occurrence, employees often resort to passive coping mechanisms. While these may temporarily alleviate internal stress and negative emotions, they fail to address the underlying issues fundamentally and may even lead to adverse outcomes. Achieving a comprehensive understanding and rational utilization of after-hours work connectivity, while maximizing its positive effects and mitigating negative impacts, has become a matter of paramount importance for both organizations and employees. However, the current studies analyzing the double-edged sword effect on its outcome variables from a comprehensive perspective mostly focus on work attitude and work–life balance. There is a relative lack of research on the mechanism of the impact of after-hours work connectivity on employee performance. Job performance is the guarantee of stable operation and goal realization of enterprises, so it is necessary to explore this path mechanism in depth.

Job crafting refers to the adjustments made by employees to better balance the demands and resources of their work with their individual abilities and needs [23]. Several studies have indicated that employees adopt passive avoidance strategies when they are exposed to significant job stress and work in high-autonomy work environments. In addition to this, they actively engage in job crafting to adapt to work pressure and protect personal resources [24]. The conservation of resources (COR) theory supports this viewpoint, indicating that when employees perceive the possibility of individual resource loss and threat, they will take measures to protect existing resources to prevent further resource depletion [25,26]. Simultaneously, they will engage in proactive actions, investing some resources to build personal resources and gain potential resource returns [27]. After-hours work connectivity is essentially a “latent” work demand, wherein employees continually experience a series of stressors, leading to the depletion of personal resources [28]. To alleviate stress and protect existing resources, employees are likely to engage in approach-oriented job crafting, such as proactively expanding job responsibilities or social relationships, taking on additional tasks, and acquiring more work-related resources [29]. Alternatively, they may resort to avoidance-oriented job crafting, such as reducing effort levels and minimizing job responsibilities or social interactions, to relax and conserve existing resources [30]. Employees who engage in proactive approach-oriented job crafting behaviors are able to access more resource support, thereby initiating a “value-added spiral” effect. This will accumulate richer resources and have a positive impact on job performance. On the other hand, employees’ avoidance-oriented job crafting can lead to a series of negative consequences, creating a vicious cycle that results in a “loss spiral” of resources, further undermining both employee and organizational overall performance. Therefore, after-hours work connectivity may have a double-edged impact on job performance, with job crafting serving as a mediator in this process. Most of the current explorations of the outcome variables of after-hours work connectivity are based on the boundary perspective and the motivational perspective. Different forms of job crafting represent differently oriented changes in employees’ adoption of work resources [31,32], which may have different impacts. Therefore, this study endeavors to open the black box of the role of after-hours work connectivity on job performance from a job crafting perspective. The psychological contract represents the organization’s commitment and support to employees, including provisions such as favorable working conditions, equitable compensation and benefits, and opportunities for career development, which can be viewed as a form of job resources [33]. According to the conservation of resources (COR) theory, an adequate supply of job resources helps individuals alleviate the impact of work stress or negative emotions, while also demonstrating more positive work attitudes and behaviors [26]. Continuous engagement in after-hours work connectivity, distinct from conventional overtime, often lacks commensurate rewards, leading to perceptions of inequity and subsequent employee dissatisfaction. When employees and organizations establish a mutually agreed-upon psychological contract, employees perceive that engaging in after-hours work connectivity may lead to greater resource support and fair compensation, potentially resulting in the adoption of positive behaviors; conversely, this may manifest in adopting passive avoidance behaviors [34]. Therefore, this study attempts to explore whether the psychological contract can moderate the relationship between after-hours work connectivity and job crafting.

In conclusion, in order to deeply explore the potential influence mechanism of after-hours work connectivity on job performance, this study constructs a parallel dual mediation model based on the conservation of resources theory. It uses approach-oriented job crafting as a facilitating mechanism and avoidance-oriented job crafting as an inhibiting mechanism. This study explores the double-edged sword effect of after-hours work connectivity on employee performance from both positive and negative perspectives as well as the moderating role of the psychological contract in the above path. This paper deeply analyzes the mechanism and boundary conditions of the role of after-hours work connectivity on employees’ job performance. The research aims to assist organizations in adopting a dialectical perspective towards and effectively utilizing after-hours work connectivity. By identifying a mutually beneficial work paradigm, this study aims to maximize the positive effects of after-hours work connectivity while mitigating its potential negative impacts, thus providing managerial recommendations to foster sustainable development for both organizations and employees.

## 2. Theoretical Foundation and Research Hypotheses

### 2.1. After-Hours Work Connectivity and Job Performance

After-hours work connectivity enables employees to maintain flexibility in staying connected with their work, enhancing autonomy and perceived control over their tasks. This fosters positive psychological experiences and job performance among employees. Moreover, prolonged work connectivity invisibly increases employees’ work engagement, as they invest more time and effort into their tasks. This heightened engagement facilitates better task completion, improved work efficiency, and prosperity within the work domain [35]. However, on the other hand, this type of work connectivity may also lead employees to continuously engage in work during non-work hours, thereby triggering work-related stress and fatigue, resulting in negative psychological experiences and job performance [36]. After-hours work connectivity gradually encroaches upon employees’ time and space, making it difficult for them to disconnect from work and have sufficient rest, thus increasing the likelihood of emotional exhaustion [13], exacerbating work fatigue [36], and fostering the emergence of negative work behaviors [17,20]. Employees may engage in avoidance and absenteeism behaviors, resulting in adverse work outcomes [18,22]. Therefore, after-hours work connectivity exhibits a typical double-edged sword effect, which may result in different impacts on employees’ job performance. Given the coexistence of advantages and disadvantages in the relationship between after-hours work connectivity and employee job performance, this study does not posit unidirectional effects.

### 2.2. Mediating Role of Job Crafting

While after-hours work connectivity has enhanced the flexibility of work-related communication to some extent [37], it also necessitates employees to be “online” around the clock, intruding into their personal lives. This requirement for continuous online presence imposes additional burdens on employees, encroaching upon their rest time and blurring the boundaries between work and personal life [7]. Moreover, excessive occupation of family time and energy for work purposes may lead to conflicts within the family unit [14,36]. As employees frequently utilize communication tools during non-working time, they increasingly rely on information and communication technologies (ICTs) for communication during working time. This trend reduces opportunities for face-to-face interaction, fostering a heightened sense of professional isolation among employees [38]. Simultaneously, frequent contact by supervisors with employees during non-work hours may lead employees to feel over-monitored or excessively demanded, resulting in psychological resistance. These factors contribute to employees continuously facing and experiencing this series of stressors, leading to the depletion of personal resources [7,39].

According to the conservation of resources theory, individual resources are finite. When employees perceive depletion or threats to their resources, they undertake corresponding measures to safeguard and replenish them, thus alleviating stress [25,40]. Employees may engage in proactive actions, investing partial resources to build individual resources and attain potential returns [25]. This may involve seeking more efficient work methods, redesigning tasks to enhance personal efficiency and performance [41], or improving interpersonal relationships within the organization by altering communication patterns. By fostering smooth collaboration and emotional connections with peers and leaders, individuals can swiftly acquire resource returns [42]. The individual’s approach-oriented job crafting behavior represents a proactive action investment in resolving work-related issues, such as proactively expanding job responsibilities or social relationships, and attempting to use new knowledge or technology to simplify work [30]. Particularly when after-hours work connectivity becomes the new norm in the workplace, employees perceive threats to their resources in this work context. According to the conservation of resources theory, employees are more likely to take proactive action to improve and design work and relationships in order to obtain possible resource rewards to build their personal resources. Simultaneously, to safeguard existing resources from further depletion, employees may also impede the continued depletion of individual resources through avoidance-oriented job crafting, such as lowering the level of work effort and reducing job duties and social relationships [30]. This behavior is an employee’s coping mechanism in response to excessive work demands or resource imbalances. By reducing personal investment, individuals aim to alleviate work pressure, mitigate feelings of unfairness, or prevent further resource depletion, thereby maintaining a balance within their work ecosystem. Based on this premise, Hypothesis 1 is proposed in this study.

**H1a.**  *After-hours work connectivity is positively related to approach-oriented job crafting.*

**H1b.**  *After-hours work connectivity is positively related to avoidance-oriented job crafting.*

Existing research generally shows that job crafting promotes job performance. However, some studies have pointed out that different types of job crafting have different impacts on job performance and that accurately distinguishing between the different dimensions of job crafting is crucial to analyzing its impact on performance. Approach-oriented job crafting involves proactive adjustments aimed at problem solving and work enhancement to achieve gains in motivation, health, and performance through modifications in job roles and social relationships. Existing research has confirmed that approach-oriented job crafting contributes to enhancing employees’ job skills and adaptability and fostering proactive engagement, resulting in positive outcomes such as person–job fit, thus achieving a win–win scenario in terms of job significance and performance [29,43]. Approach-oriented job crafting primarily manifests in seeking work resources and challenging job demands, such as actively expanding job responsibilities or social relationships, organizing tasks systematically, and utilizing new knowledge or technology to streamline work processes. Expanding job responsibilities refers to employees taking on tasks beyond the formal job design, from which they can derive meaning [30]. For instance, when employees engage in work-related activities during non-working time, actively participating in team discussions, voicing opinions on essential matters, and providing strategic suggestions can enhance their sense of meaningfulness in work [44]. Acquisition of these psychological resources can inherently motivate employees to strive harder, positively impacting their job performance [45]. Expanding social relationships refers to employees proactively establishing connections with others to gain self-worth and garner respect from peers [30]. Employees may utilize their non-working hours to explain product features and considerations to clients in order to earn their recognition. Furthermore, employees may also enhance work efficiency by strategically organizing tasks, devising novel work methods, or employing new technologies to streamline workflow, thereby reducing after-hours work connectivity. In conclusion, through approach-oriented job crafting, employees can acquire more support resources. This includes enhancing their job skills, increasing autonomy and control; fulfilling competency needs; and establishing mutual trust and mutually beneficial interpersonal relationships with colleagues, supervisors, and clients. Consequently, this fosters individuals’ full utilization of subjective initiative, enhances intrinsic motivation, facilitates better task performance, and improves job performance [29,31,46,47].

Avoidance-oriented job crafting refers to a passive form of job adjustment characterized by defensive, avoidance, and withdrawal behaviors, where employees, driven by the desire to minimize personal resource depletion, adjust their job roles and social relationships. Existing research indicates that avoidance-oriented job crafting may lead to a lack of initiative when facing tasks, resulting in a reluctance to proactively address issues, thereby impacting work efficiency and productivity [29,30]. Avoidance-oriented job crafting is primarily manifested in reducing obstructive work demands, such as diminishing work roles and social interactions [48]. Reducing job roles involves minimizing tasks beyond job design, which leads individuals to balance resources allocated for task cognition and completion. As individuals reduce their work engagement to alleviate pressure, resources for task reflection and completion decrease, potentially resulting in diminished task quality, errors, or omissions, adversely affecting job performance [29,30,32]. Reducing social relationships entails employees avoiding interactions with supervisors, colleagues, and clients, hindering the establishment of effective cooperation and emotional connections in the workplace, thereby undermining the enhancement of task efficiency. In summary, while avoidance-oriented job crafting may facilitate the restoration of employees’ mental, emotional, or physical resources, it also deprives them of the opportunity to reinvest resources to enhance performance levels, thereby exerting a negative impact on job performance [49]. Based on this premise, Hypothesis 2 is proposed in this study.

**H2a.**  *Approach-oriented job crafting is positively related to job performance.*

**H2b.**  *Avoidance-oriented job crafting is negatively related to job performance.*

In summary, faced with the work context where after-hours work connectivity leads to a series of stressors resulting in individual resource depletion among employees, individuals will actively seek more work resources and avoid further loss of their resources [25]. On the one hand, employees may adopt approach-oriented job crafting strategies, such as voluntarily expanding job responsibilities or strengthening social connections, to actively seek and acquire more work resources. Alternatively, they may explore adaptive strategies [26,41]. On the other hand, employees may adopt a defensive mode by engaging in avoidance-oriented job crafting to preserve existing resources. This can involve reducing work engagement, minimizing responsibilities, or scaling back social activities to prevent further resource depletion [30]. Through adopting proactive approach-oriented job crafting behaviors, employees can acquire additional resource support, thus initiating a “value-added spiral” effect, ultimately accumulating a more abundant total resource pool. Through avoidance-oriented job crafting, employees may miss out on opportunities for learning and growth, leading to imbalanced workloads and potentially forcing other team members to assume tasks avoided by the employee, causing dissatisfaction among colleagues. Consequently, a series of negative impacts may ensue, forming a vicious cycle and plunging into a spiral of resource loss, further weakening the overall performance of the employee and the organization. Based on this premise, Hypothesis 3 is proposed in this study.

**H3a.**  *Approach-oriented job crafting mediates the relationship between after-hours work connectivity and job performance, indicating that after-hours work connectivity positively predicts job performance through approach-oriented job crafting.*

**H3b.**  *Avoidance-oriented job crafting mediates the relationship between after-hours work connectivity and job performance, indicating that after-hours work connectivity negatively predicts job performance through avoidance-oriented job crafting.*

### 2.3. The Moderating Role of the Psychological Contract

The psychological contract refers to employees’ subjective expectations or understandings of the obligations and responsibilities that organizations should fulfill in labor relations [50]. The psychological contract at different levels serves as a condition of the work environment and an individual resource, influencing employees’ behavioral choices in the context of after-hours work connectivity [51,52]. According to the conservation of resources theory, employees with sufficient resources will show more positive work attitudes. When the psychological contract is perceived to be high, it indicates that employees believe that they are supported and rewarded with adequate resources when they engage in work connectivity behaviors during non-work time. At this time, employees are more likely to adopt proactive behaviors such as approach-oriented job crafting to acquire and create resources, thereby expanding their resource pool [26]. Following the “value-added spiral” concept within the conservation of resources (COR) theory [25], when organizations provide employees with an ample supply of resources necessary for personal development, employees are more motivated to make adjustments and improvements. Therefore, when employees perceive that engaging in work-related tasks during non-work time will result in additional resource support and fair compensation, along with an increase in emotional resources, they are more inclined to demonstrate proactive behaviors. Through approach-oriented job crafting, employees enhance structural job resources, such as actively learning new knowledge and skills, innovatively considering work methods, proposing improvement suggestions, or investing additional physical or psychological resources to undertake extra work responsibilities [53].

Conversely, employees with fewer resources, due to experiencing high levels of stress and energy depletion, may become trapped in a “spiral of loss,” resulting in a decrease in their overall resource pool [25]. When the level of perceived psychological contract is low, employees perceive that the organization is not providing them with adequate resource support and reasonable compensation in return. These employees may feel that their resources are continuously diminishing when faced with after-hours work connectivity [54,55]. They develop a sense of unfairness and reduce their identification with the organization [56]. Consequently, the continuous loss of resources prompts employees to enter a defensive state, where the resources they allocate for thinking and executing work tasks gradually diminish. In such circumstances, employees may require more time and resources to restore their work efficiency and patterns to normal. To stop further depletion of resources, they may be more inclined to employ passive behaviors, such as avoidance-oriented job crafting, to cope with work tasks. In light of this, Hypothesis 4 is proposed in this study.

**H4a.**  *The psychological contract positively moderates the relationship between after-hours work connectivity and approach-oriented job crafting, such that after-hours work connectivity has a stronger positive relationship with approach-oriented job crafting under a high psychological contract.*

**H4b.**  *The psychological contract negatively moderates the relationship between after-hours work connectivity and avoidance-oriented job crafting, such that after-hours work connectivity has a stronger negative relationship with avoidance-oriented job crafting under a low psychological contract.*

Based on the analysis mentioned above, this study further posits that the moderating effect of the psychological contract on the mediating role of work intrusion into non-work time and job performance may also exist. Specifically, compared to employees with low psychological contract perceptions, those perceiving high psychological contract tendencies are more inclined to engage in approach-oriented job crafting behaviors. When employees perceive a higher level of the psychological contract, based on the initial resource effect, individuals with richer initial resources are less susceptible to resource loss and more likely to acquire new resources [26]. In this context, employees perceiving high levels of psychological contract are more likely to actively invest personal resources and take risks when facing work intrusion into non-work time, thereby exhibiting proactive behaviors and enhancing job performance. Conversely, employees perceiving lower levels of the psychological contract, when confronted with after-hours work connectivity, perceive their resources as being continuously depleted, resulting in increased feelings of insecurity and unfairness. Consequently, they initiate defensive mechanisms, reducing work engagement, engaging in avoidance-oriented job crafting behaviors, and diminishing job performance. Based on this, when psychological contracts are high, the positive impact of after-hours work connectivity on employees’ job performance through approach-oriented job crafting is stronger. Consequently, approach-oriented job crafting more effectively mediates the positive effects of after-hours work connectivity on employees’ job performance [29]. Conversely, when psychological contracts are low, employees tend to utilize avoidance-oriented job crafting to cope with after-hours work connectivity, exacerbating the negative effects of after-hours work connectivity on job performance [30]. Thus, Hypothesis 5 is proposed in this study.

**H5a.**  *The psychological contract positively moderates the mediating effect of approach-oriented job crafting between after-hours work connectivity and job performance. The higher the level of the psychological contract, the stronger the mediating effect of approach-oriented job crafting between after-hours work connectivity and job performance.*

**H5b.**  *The psychological contract negatively moderates the mediating effect of avoidance-oriented job crafting between after-hours work connectivity and job performance. The lower the level of the psychological contract, the stronger the mediating effect of avoidance-oriented job crafting between after-hours work connectivity and job performance.*

**Figure 1:**
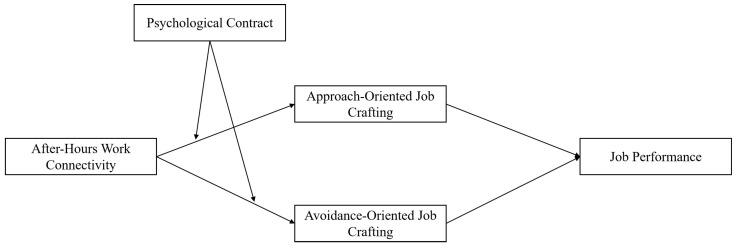
Theoretical model diagram.

Based on this, the hypothesis model of this study is illustrated in Figure 1.

## 3. Methods

### 3.1. Sample and Procedure

Considering that full-time employees have more regular and defined working hours and responsibilities in the company, their non-work time patterns are relatively consistent. We employed a questionnaire survey to collect data, targeting full-time employees of enterprises with fixed working hours in the Jiangsu, Anhui, Shanghai, Zhejiang, and Shandong provinces. A combined online and offline approach was adopted for questionnaire data collection. Offline surveys were distributed on-site at internship units and neighboring companies, with staff guiding completion of the questionnaires. Online surveys were disseminated using the Questionnaire Star platform, leveraging connections with friends and family already in employment to assist in questionnaire completion. Through their social networks, respondents were encouraged to forward the questionnaire to their colleagues, thus expanding the survey’s coverage. The survey was conducted from January to March 2024, during which a total of 600 questionnaires were distributed. Of these, 517 were returned, resulting in a response rate of 86.17%. Questionnaires with response times of less than 80 s, identical responses, or significant logical errors were considered invalid. Ultimately, 407 valid questionnaire responses were collected, yielding an effective response rate of 78.72%.

Demographic characteristics of the respondents were as follows: in terms of gender distribution, males accounted for 51.4%, and females accounted for 48.6%. In terms of age distribution, the proportion of individuals ages 25 and below was 39.1%, while those ages 26–35 constituted 28%. Individuals ages 36–45 represented 18.4% of the population, whereas those ages 46 and above accounted for 14.5%. Regarding educational attainment, the proportion of individuals with a high school (or vocational school) education or below was 15.2%. Those with a college degree comprised 16.7% of the population, while individuals with a bachelor’s degree represented 40.8%. Furthermore, those with a master’s degree or higher constituted 27.3% of the population. In terms of variation in actual work experience, the proportion of individuals with less than one year of experience was 34.1%, while those with 1–5 years of experience made up 44.7% of the population. Individuals with 6–10 years of experience represented 10.1%, and those with more than 10 years of experience accounted for 11.1%. Regarding hierarchical distribution within the workforce, ordinary employees made up 63.7% of the population, while front-line managers represented 18.9%. Middle managers constituted 13.5%, and senior executives comprised 3.9% of the population.

### 3.2. Measurement Instrument

To validate the research hypotheses, this study employed the Likert 5-point scale to measure relevant variables and conducted empirical tests on the data obtained from the questionnaire survey. The questionnaire items are detailed in Appendix A.

1. After-hours work connectivity: In this study, measurement was conducted using a scale developed by Ma et al. [57] comprising three items. This new scale is suitable for the local context in China and has been widely cited by domestic scholars. A typical example of a question is, “During non-work time (before work, during lunch breaks, after work, and on weekends and holidays), individuals related to work contact me via phone calls, emails, or messages (such as DingTalk, WeChat, Feishu)”. The Cronbach’s α of this scale was 0.863.
2. Job crafting: This study primarily referenced Bruning and Campion’s definition of job crafting and their delineation of dimensions. Therefore, the Work Crafting Scale developed by Bruning and Campion [30] was utilized for the empirical analysis to ensure the reliability of the research findings. Given that this study leans towards understanding job crafting from a behavioral perspective, we excluded items related to metacognitive dimensions from the scale, resulting in a final set of 25 items. Among these, there were 18 items for approach-oriented job crafting and 7 items for avoidance-oriented job crafting, with a representative item being “I express my opinions on important issues and provide suggestions for the team or organization”. In this study, the Cronbach’s α for approach-oriented job crafting was 0.932, while for avoidance-oriented job crafting, it was 0.873.
3. Job performance: The “Task-Context” two-dimensional model scale developed by Borman et al. [58] has been widely adopted by numerous scholars for exhibiting good reliability and validity. This scale not only measures employees’ ability levels in completing tasks but also includes a series of positive behaviors related to achieving work objectives. Recognizing the two-dimensional structure of job performance, this study utilized the work performance scale developed by Borman et al. [58] for empirical analysis. The scale comprises 12 items, with a representative item being “I am consistently able to complete tasks within the allotted time”. In this study, the Cronbach’s α for this scale was 0.920.
4. Psychological contract: The psychological contract scale developed by Li and Guo [59], based on the actual situation in China, has been widely adopted by domestic scholars. Numerous empirical studies have confirmed the scale’s high reliability and validity. Given that this study regards the psychological contract as employees’ subjective expectations or understandings of the obligations and responsibilities that organizations should fulfill in labor relations, the scale’s “organizational obligations” dimension was employed for measurement, comprising a total of 13 items. A representative item included, “The organization provides me with a safe and comfortable working environment”. In this study, the Cronbach’s α of the scale was found to be 0.912.
5. Control variables: Gender and age are typically associated with differences in attitudes, values, and preferences since they can impact employees’ work behaviors and outcomes [60,61]. Education, background, and tenure are related to knowledge, skills, and experience, which also influence job performance [62,63]. Additionally, different ranks may affect employees’ job crafting strategies, ultimately impacting job outcomes [64]. Therefore, to ensure the accuracy of the results, this study includes employees’ demographic characteristics—gender, age, highest education level, tenure, and job rank—as control variables for analysis.

### 3.3. Data Analysis

We analyzed the collected data set by employing reliability, validity, and correlations using SPSS 27.0 and AMOS 26.0 software packages. Cronbach’s α coefficients were computed to establish the reliability of the scales; a Cronbach’s alpha coefficient exceeding 0.8 indicates that the scale has high stability and reliability. Then, we conducted descriptive statistics for the variables and used SPSS 27.0 to calculate the Pearson correlations among the research variables. Subsequently, confirmatory factor analysis was conducted using AMOS 26.0 software. Before analysis, variables with more than three items were grouped using the parceling measurement approach proposed by Wu and Wen [65]. To evaluate the fit of the proposed research model, the χ2 statistic was utilized along with the comparative fit index (CFI), Tucker–Lewis index (TLI), incremental fit index (IFI), and root mean square error of approximation (RMSEA). χ2/df value less than 3; CFI, IFI, and TLI values greater than 0.9; and an RMSEA value less than 0.08 indicate a good model fit. Furthermore, to test the research hypotheses, we adopted the mediation analysis method proposed by Preacher and Hayes [66], utilizing the process macro and the bootstrap approach to examine the mediating effects of approach-oriented job crafting and avoidance-oriented job crafting. The bootstrap method was employed for the mediation analysis, with 5000 bootstrap samples, 95% confidence intervals, and bias-corrected non-parametric percentile method sampling. The direct effects of each variable’s path coefficients were analyzed, and the significance of the indirect effects was tested. According to the criteria, if the bootstrap confidence interval does not include a zero value, it indicates a significant mediating effect; otherwise, it is considered non-significant. The moderation effects of the psychological contract were examined using hierarchical regression analysis. The level of statistical significance was set at *p* < 0.05.

## 4. Results

### 4.1. Discriminant Validity Analysis and Common Method Bias Test

This study employed AMOS 26.0 software for confirmatory factor analysis. Table 1 presents the results of the confirmatory factor analysis, indicating that the χ2/df ratio is 1.833, which is less than 3. Additionally, the values of CFI, IFI, and TLI are 0.974, 0.975, and 0.965, respectively, all exceeding 0.9, while the RMSEA value of 0.045 is less than 0.08. These results suggest that the five-factor model fits well and outperforms alternative models, which suggests that all five major variables exhibit robust construct validity.

As can be seen in Table 2, the AVE square root values of the five variables of after-hours work connectivity, approach-oriented job crafting, avoidance-oriented job crafting, job performance, and psychological contract are greater than the correlation coefficients between any of the variables. This indicates that there is a discriminant validity among the variables.

As all measurements of the variables were self-reported by respondents, common method variance may exist. Furthermore, this study conducted Harman’s single-factor test on the 53 items related to the five variables. The analysis revealed that the cumulative variance explained by the first factor is 25.929%, below the empirical standard of 40%. This indicates that the data are not significantly affected by serious common method bias, thus validating the effectiveness and credibility of the research findings.

### 4.2. Descriptive Statistics and Correlation Analysis

Before conducting the regression analysis, descriptive statistics and the correlation analysis were performed using SPSS 26.0 for the variables involved in this study, as shown in Table 3. The results indicate significant positive correlations between after-hours work connectivity and approach-oriented job crafting (r = 0.355, *p* < 0.01) as well as between approach-oriented job crafting behavior and job performance (r = 0.337, *p* < 0.01). Furthermore, after-hours work connectivity is significantly positively correlated with avoidance-oriented job crafting (r = 0.134, *p* < 0.01). In contrast, avoidance-oriented job crafting behavior exhibits a significant negative correlation with job performance (r = −0.261, *p* < 0.01). These correlation analysis findings provide initial support for the research hypotheses and lay the groundwork for further data analyses.

### 4.3. Hypothesis Testing

#### 4.3.1. Mediation Effect Testing

**Figure 2:**
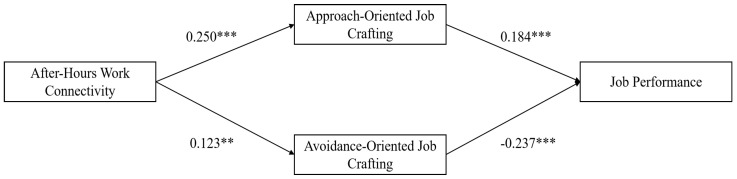
Path analysis results. *** *p* < 0.001, ** *p* < 0.01.

Drawing on the research by Preacher and Hayes [66], the mediation effects of approach-oriented job crafting and avoidance-oriented job crafting were examined using the process program and bootstrap method. Specifically, Model 4 of the process program was utilized with 5000 bootstrap resamples. The control variables of after-hours work connectivity, approach-oriented job crafting, avoidance-oriented job crafting, and job performance were simultaneously included in the analysis. The results of the path analysis are presented in Table 4 and Figure 2, while the indirect effects are displayed in Table 5. From the results, it is observed that after-hours work connectivity positively predicts approach-oriented job crafting (β = 0.250, *p* < 0.001), supporting Hypothesis 1a. Approach-oriented job crafting is significantly positively associated with job performance (β = 0.184, *p* < 0.001), supporting Hypothesis 2a. The indirect effect of after-hours work connectivity on job performance through approach-oriented job crafting is 0.046, with a 95% confidence interval of [0.017, 0.077]. Since the interval does not contain zero, it indicates a significant indirect effect of approach-oriented job crafting, supporting Hypothesis 3a.

Additionally, after-hours work connectivity positively predicts avoidance-oriented job crafting (β = 0.123, *p* < 0.01), supporting Hypothesis 1b. Avoidance-oriented job crafting is significantly negatively correlated with job performance (β = −0.237, *p* < 0.001), supporting Hypothesis 2b. The indirect effect of after-hours work connectivity on job performance through avoidance-oriented job crafting is −0.029, with a 95% confidence interval of [−0.005, −0.008]. Since the interval does not include zero, it indicates a significant indirect effect of avoidance-oriented job crafting, supporting Hypothesis 3b.

#### 4.3.2. Moderation Effect Testing

The moderation effect of psychological contract was examined using hierarchical regression analysis. Prior to the analysis, after-hours work connectivity and psychological contract were centered, and their interaction term was constructed. The regression analysis results are presented in Table 6, indicating that the interaction term between after-hours work connectivity and psychological contract significantly influences approach-oriented job crafting (β = 0.142, *p* < 0.01) and avoidance-oriented job crafting (β = −0.155, *p* < 0.01). Specifically, psychological contract positively moderates the relationship between after-hours work connectivity and approach-oriented job crafting, while significantly negatively moderating the relationship between after-hours work connectivity and avoidance-oriented job crafting.

**Figure 3:**
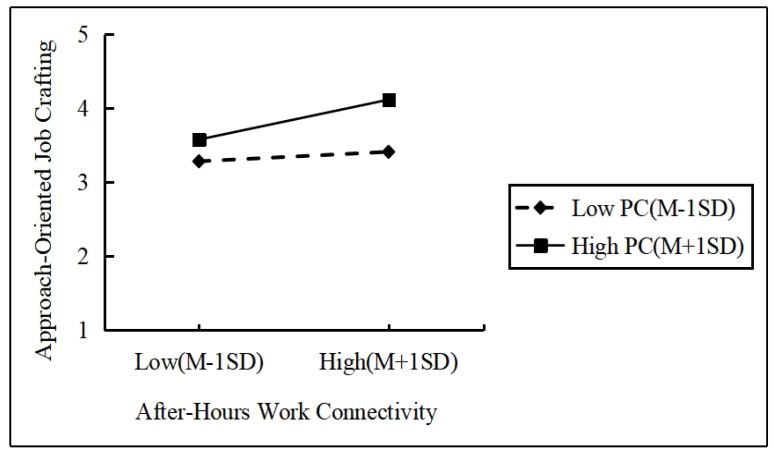
Map of the moderating effect of the psychological contract.

**Figure 4:**
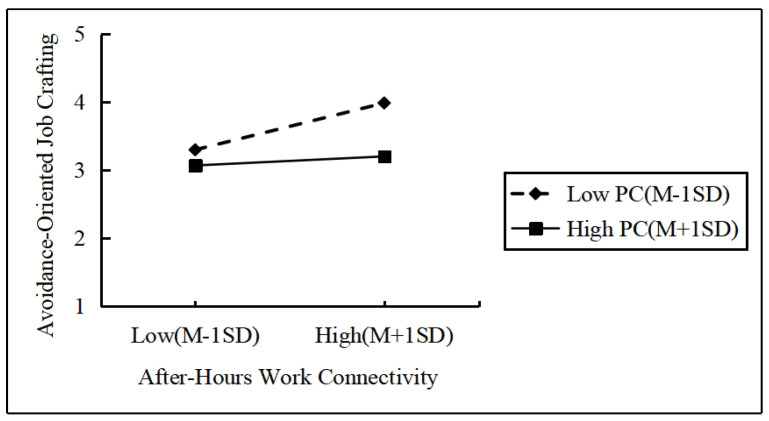
Map of the moderating effect of the psychological contract.

To visually illustrate the moderating effect of the psychological contract on the relationship between after-hours work connectivity and approach-oriented job crafting, this study plotted the effects of after-hours work connectivity on approach-oriented job crafting and avoidance-oriented job crafting at high and low levels of psychological contract, as shown in Figure 3 and Figure 4. From the figures, it can be observed that when the psychological contract is at a lower level, the slope of the effect of after-hours work connectivity on approach-oriented job crafting is relatively gentle, while the slope on avoidance-oriented job crafting is steeper. Conversely, when the psychological contract is at a higher level, the slope of the effect of after-hours work connectivity on approach-oriented job crafting becomes steeper, whereas the slope on avoidance-oriented job crafting becomes gentler. This indicates that as the level of psychological contract increases, the positive impact of after-hours work connectivity on approach-oriented job crafting intensifies, whereas at lower levels of psychological contract, the effect of after-hours work connectivity on avoidance-oriented job crafting becomes more pronounced. This further validates the positive moderating effect of psychological contract on the relationship between non-work time job connectivity and approach-oriented job crafting as well as the negative moderating effect on the relationship between after-hours work connectivity and avoidance-oriented job crafting.

#### 4.3.3. Moderated Mediation Effect Testing

Additionally, Table 7 displays the differences in the mediating effects of approach-oriented job crafting and avoidance-oriented job crafting between after-hours work connectivity and job performance at different levels of the psychological contract. Under the low-level psychological contract, the indirect effect of approach-oriented job crafting in the relationship between after-hours work connectivity and job performance is relatively low, with an indirect effect of 0.012 and a 95% confidence interval of [−0.008, 0.034], which includes zero, indicating non-significance. Conversely, under the high-level psychological contract, the indirect effect of approach-oriented job crafting on the relationship between after-hours work connectivity and job performance is relatively high, with an indirect effect of 0.050 and a 95% confidence interval of [0.018, 0.087], which does not include zero, indicating significance. Furthermore, there is a significant difference in the indirect effects between the two levels, with a difference of 0.038 and a 95% confidence interval of [0.009, 0.079], which does not include zero. Overall, there exists a moderated mediation effect, indicating that the indirect effect of after-hours work connectivity on job performance through approach-oriented job crafting varies. Specifically, the higher the level of the psychological contract, the stronger the indirect effect of after-hours work connectivity through approach-oriented job crafting on job performance, supporting Hypothesis 5a. Under the low-level psychological contract, the indirect effect of avoidance-oriented job crafting in the relationship between after-hours work connectivity and job performance is relatively high, with an indirect effect of −0.082 and a 95% confidence interval of [−0.127, −0.044], which does not include zero, indicating significance. Conversely, under the high-level psychological contract, the indirect effect of avoidance-oriented job crafting in the relationship between after-hours work connectivity and job performance is relatively low, with an indirect effect of −0.016 and a 95% confidence interval of [−0.045, 0.012], which includes zero, indicating non-significance. Furthermore, there is a significant difference in the indirect effects between the two levels, with a difference of 0.066 and a 95% confidence interval of [0.024, 0.117], which does not include zero. In summary, there exists a moderated mediation effect, indicating that the indirect effect of after-hours work connectivity on job performance through avoidance-oriented job crafting varies. Specifically, the lower the level of psychological contract, the stronger the indirect effect of after-hours work connectivity through avoidance-oriented job crafting on job performance, providing support for Hypothesis 5b.

## 5. Discussion

The research findings indicate the following: (1) Proactive pathway: after-hours work connectivity tends to reshape work positively, predicting work performance. When employees engage in continuous non-work-related connectivity, experiencing resource depletion, they tend to adopt approach-oriented job crafting to invest resources in acquiring more, thereby positively affecting job performance. (2) Passive pathway: after-hours work connectivity negatively predicts job performance through avoidance-oriented job crafting. Faced with sustained connectivity-induced resource losses, employees tend to adopt avoidance-oriented job crafting to prevent further resource depletion, thus adversely affecting job performance. (3) Psychological contracts play a positive moderating role between after-hours work connectivity and approach-oriented job crafting while playing a negative moderating role between after-hours work connectivity and avoidance-oriented job crafting. Additionally, the moderating effect of psychological contracts on the mediated path is verified. When the level of psychological contract is higher, employees are more motivated to adopt approach-oriented job crafting behavior, which further promotes resource acquisition and performance gains. Conversely, it triggers a defensive state, making employees more inclined to adopt avoidance-oriented job crafting behavior, thereby negatively impacting performance.

### 5.1. Theoretical Contribution

This study, starting from the perspective of employees’ coping strategies, explores the mechanism through which after-hours work connectivity influences job performance. This study enriches the research on the subsequent impact of after-hours work connectivity on the workplace. Most previous studies have focused on exploring its impact on individual psychological aspects (such as enhancing autonomy and sense of control [7,8]) and life domains (such as work–family enrichment [28]) and have to some extent neglected the outcome variables in the work domains of interest to firms. In addition, this study further extends the research on the positive path effects of after-hours work connectivity. Existing studies have mainly focused on the impact of after-hours work connectivity on employees’ negative work attitudes and behaviors [7,14] and have argued that after-hours work connectivity is a latent job demand that can have adverse work consequences [17,18]. In order to understand its positive impact on job performance outcomes, this study was further explored. Abundant literature demonstrates that when employees experience high levels of work pressure and possess greater autonomy in their job context, it tends to trigger them to engage in job crafting [23,67]. Positive work redesign behaviors have been shown to positively influence work performance [29,30]. Therefore, this paper introduces approach-oriented job crafting as a mediator and explores the positive path of the impact of after-hours work connectivity on job performance. Faced with a series of stressors resulting from after-hours work connectivity, employees can also adopt proactive coping measures such as approach-oriented job crafting to reverse the situation and mitigate stress, leading to positive work outcomes. This provides a more comprehensive and enriched research perspective on after-hours work connectivity, job crafting, and job performance theories, also responding to the call by Park et al. [68] for increased research on the positive effects of work connectivity behavior.

Based on the conservation of resources theory, this study adopts a comprehensive perspective to elucidate the underlying mechanisms between after-hours work connectivity and job performance. This research constructs a dual-path model of approach-oriented job crafting and avoidance-oriented job crafting, explaining the discrepancies in conclusions drawn from a singular perspective in studying after-hours work connectivity. After-hours work connectivity, as a new norm in the workplace under the backdrop of technological empowerment, harbors both benefits and drawbacks [10,16]. Previous domestic and international studies have often focused on elucidating the mechanisms of after-hours work connectivity from a singular perspective of either positivity or negativity [7,11,69]. A few scholars have recently adopted a comprehensive perspective to dialectically examine the dual-edged sword effect of after-hours work connectivity [19,28]. However, limited research has primarily focused on exploring the impact of after-hours work connectivity on employees’ work attitudes and behaviors [17,19], leaving a relative lack of analysis on the pathway mechanisms through which after-hours work connectivity affects individual performance. Adopting a dialectical perspective on the relationship between the variables, this study elucidates how after-hours work connectivity leads to resource depletion and how it promotes resource investment (approach-oriented job crafting) and resource conservation (avoidance-oriented job crafting), which ultimately affects job performance. This study integrates the pros and cons of after-hours work connectivity for both employees and organizations and explores the impact of this behavior on employee performance in a more comprehensive and dialectical manner.

This study elucidates the boundary conditions of the mechanism through which after-hours work connectivity affects employees’ job performance, particularly by introducing the psychological contract as a moderating variable. Existing research has indicated that psychological contracts affect numerous employment outcomes, both in terms of their content and the extent to which they are fulfilled or violated [51,52]. They can influence final performance outcomes by affecting employees’ work attitudes and behaviors [54]. For employees, after-hours work connectivity represents an irresistible form of “implicit overtime” job demand [28]. The attitudes and behaviors that employees exhibit when facing after-hours work connectivity depend on their psychological perceptions of it [11]. Existing literature has confirmed that employees’ psychological states can influence the effectiveness of after-hours work connectivity implementation [70]. The psychological contract, as a tacit, trust-based form of agreement, tends to stimulate employees’ positive work attitudes and mitigate potential negative impacts when effectively constructed and maintained. In such instances, employees are more likely to perceive investing non-work time into work as a mutual cooperation. Conversely, breach of the psychological contract can elicit employees’ negative work attitudes and behaviors. This study extends the boundary conditions of the impact mechanism of after-hours work connectivity, providing guidance for effectively leveraging its positive effects and mitigating negative effects.

### 5.2. Practical Implications

After-hours work connectivity resembles a double-edged sword, bearing both benefits and drawbacks. With the widespread application of information and communication technologies in the workplace, the modes of communication and interaction among employees have transformed. After-hours work connectivity has thus become an unavoidable work habit in organizations, leading to pressures for adjustments in organizational management practices. The immediate priority for enterprises is to utilize effective management strategies, employing astute tactics to harness its benefits, mitigate its drawbacks, and achieve more positive application outcomes, thereby enhancing overall employee and organizational performance. This study provides the following recommendations for organizational management as a reference.

Firstly, managers should pay attention to the negative effects of after-hours work connectivity to avoid employees adopting passive and negative ways to cope with pressure and avoid problems. When implementing after-hours work connectivity, organizations should pay attention to the frequency and duration of communication, and the after-hours work connectivity with employees should be moderate to ensure the “right to be offline” and “right to be disconnected” of employees and to ensure that employees have enough non-work time for rest and personal life. Managers need to fully understand the workload and pressure on employees and provide policy options to support work–life balance, such as telecommuting and flexible working hours, in order to reduce the work pressure of employees and prevent them from adopting avoidance-oriented job crafting.

Secondly, managers should fully explore the positive effects of after-hours work connectivity and guide employees to take a proactive approach to challenges. Organizations can shape a corporate culture that encourages innovation, trial and error, and proactive problem solving while providing a resource support mechanism to maintain employee enthusiasm and engagement to encourage the adoption of approach-oriented job crafting. Managers can create an environment where each employee is given a degree of job adjustments. This includes encouraging employees to participate in job design and providing support and resources to stimulate motivation and initiative.

Finally, organizations should know that the psychological contract can promote different behavioral performances of employees and should effectively manage and maintain the psychological contract to promote long-term cooperation and a win–win situation between the organization and employees. Organizations need to establish transparent and open communication channels to ensure that employees have a clear understanding of the organization’s expectations and commitments through regular communication meetings, feedback mechanisms, and information sharing. Managers can care for employees in many ways, both materially and spiritually, and provide targeted support for their personal development needs in order to increase employee identification with the organization. Organizations also need to recognize that the psychological contract is dynamic, and adjust and update the psychological contract in a timely manner when employees’ roles change to reduce the risk of psychological contract violation and better maintain the relationship between employees and the organization.

### 5.3. Limitations and Future Research

While this study endeavors to be rigorous, there are still some limitations. Firstly, in terms of questionnaire design and data collection, all variables in this study were measured using self-reported scales by employees. Due to factors such as self-awareness, employees may have been inclined to withhold information when filling out the questionnaire. In the future, employing multi-source evaluation methods for data collection could be considered. Secondly, regarding the research content, this study primarily distinguishes work reshaping into two dimensions: approach-oriented work reshaping and avoidance-oriented work reshaping. These two types of work reshaping could be further subdivided into different dimensions. Further investigation is needed to determine whether these different dimensions have significant differences in their impact on other key variables explored in this study. This will deepen the understanding of the specific mechanisms of work reshaping behaviors. Once again, a dynamic perspective is needed when exploring after-hours work connectivity. The current study, grounded in a static viewpoint while mitigating the effects of common method bias, fails to consider the longitudinal changes to the variable of after-hours work connectivity and its underlying mechanisms over time. Therefore, employing a dynamic perspective to collect and analyze data from different time points is crucial for comprehending the temporal fluctuations in individuals’ perceptions of after-hours work connectivity and its longitudinal effects. Building dynamic models of its effects represents a necessary direction for future research. Finally, this study lacked an exploration of respondents’ characteristics, such as the type of industry and the unique cultures of different countries. Attitudes toward after-hours work connectivity and job crafting may vary across industries (such as manufacturing, IT, and service industries) or among employees in different cultures. Future studies may select employees in fixed industries or other cultural contexts to further validate the findings of this study.

## 6. Conclusions

This study innovatively explores the mechanism of the relationship between after-hours work connectivity and employee job performance from the perspective of job crafting. After-hours work connectivity produces different job performance outcomes through two pathways, namely the proactive pathway and the passive pathway. In the proactive pathway, employees facing work connectivity adopt approach-oriented job crafting to increase resource acquisition and improve job performance. Conversely, in the passive pathway, employees adopt avoidance-oriented job crafting behaviors when faced with after-hours work connectivity, which has a negative effect on job performance. Additionally, the study finds that psychological contracts play a moderating role in this relationship, significantly influencing both the proactive and passive pathways. Employees with a high level of psychological contract adopt approach-oriented job crafting behaviors that further promote resource acquisition and job performance. Meanwhile, employees with a low level of psychological contract exhibit a defensive state and adopt avoidance-oriented job crafting behaviors that produce negative job performance outcomes. This study enriches and expands the empirical research on the relationships between after-hours work connectivity, job crafting, psychological contracts, and job performance. Moreover, in practice, it can assist organizations in dialectically understanding and effectively utilizing after-hours work connectivity, balancing organizational benefits and employee well-being through effective management and maintenance of psychological contracts and seeking a win–win work mode that maximizes the positive effects of after-hours work connectivity while mitigating its potential negative effects.

## Figures and Tables

**Table 1:** Comparison of fitting results between five-factor model and competition model.

Model	Factor	χ2/df	RMSEA	CFI	IFI
Five-factor model	AHWC, APJC, AVJC, JP, PC	1.833	0.045	0.974	0.975
Four-factor model	AHWC, APJC + AVJC, JP, PC	4.720	0.096	0.879	0.880
Three-factor model	AHWC, APJC + AVJC + PC, JP	7.224	0.124	0.790	0.791
Two-factor model	AHWC + APJC + AVJC + PC, JP	12.768	0.170	0.591	0.594
Single-factor model	AHWC + APJC + AVJC + JP + PC	13.767	0.177	0.551	0.554

Note: AHWC: after-hours work connectivity; APJC: approach-oriented job crafting; AVJC: avoidance-oriented job crafting; JP: job performance; PC: psychological contract.

**Table 2:** Discriminant validity test results.

Variables	AHWC	APJC	AVJC	JP	PC
AHWC	(0.688)				
APJC	0.280 ***	(0.556)			
AVJC	0.141 **	−0.094 **	(0.575)		
JP	0.306 **	0.178 ***	−0.154 ***	(0.568)	
PC	0.300 ***	0.241 ***	−0.124 ***	0.251 ***	(0.520)
AVE Square Root	0.829	0.746	0.758	0.754	0.721

Note: AHWC: after-hours work connectivity; APJC: approach-oriented job crafting; AVJC: avoidance-oriented job crafting; JP: job performance; PC: psychological contract. *** *p* < 0.001, ** *p* < 0.01; the diagonal line is the VAE value.

**Table 3:** Correlation coefficient, mean value, and standard deviation of each variable.

Variables	1	2	3	4	5	6	7	8	9	10
1. Gender	1									
2. Age	−0.002	1								
3. Education Level	−0.013	−0.204 **	1							
4. Length of Service	−0.095	0.489 **	−0.207 **	1						
5. Job Rank	−0.127 *	0.295 **	−0.046	0.304 **	1					
6. AHWC	−0.044	0.072	−0.091	0.083	0.147 **	1				
7. APJC	0.012	0.004	0.060	0.057	0.161 **	0.355 **	1			
8. AVJC	0.014	−0.030	−0.065	0.027	−0.041	0.134 **	−0.169 **	1		
9. JP	−0.024	−0.016	0.121 *	0.004	0.056	0.339 **	0.337 **	−0.261 **	1	
10. PC	0.050	0.118 *	−0.049	0.134 **	0.143 **	0.346 **	0.439 **	−0.208 **	0.416 **	1
Mean	1.490	2.080	2.800	1.980	1.580	3.217	3.632	3.343	3.551	3.250
Standard Deviation	0.500	1.073	1.006	0.941	0.867	0.995	0.714	0.883	0.773	0.679

Note: AHWC: after-hours work connectivity; APJC: approach-oriented job crafting; AVJC: avoidance-oriented job crafting; JP: job performance; PC: psychological contract. ** *p* < 0.01, * *p* < 0.05.

**Table 4:** Path coefficient results.

Action Path	Path Coefficient	BootSE	BootLLCI	BootULCI
AHWC→APJC	0.250 ***	0.034	0.184	0.316
AHWC→AVJC	0.123 **	0.044	0.036	0.210
APJC→JP	0.184 ***	0.052	0.081	0.287
AVJC→JP	−0.237 ***	0.040	−0.315	−0.160

Note: AHWC: after-hours work connectivity; APJC: approach-oriented job crafting; AVJC: avoidance-oriented job crafting; JP: job performance. *** *p* < 0.001, ** *p* < 0.01.

**Table 5:** Results of the multiple mediating effects test.

Mediation Path	Effect	BootSE	BootLLCI	BootULCI
Ind1: AHWC→APJC→JP	0.046	0.016	0.017	0.077
Ind2: AHWC→AVJC→JP	−0.029	0.012	−0.005	−0.008
Total mediation effect: Ind1 + Ind2	0.017	0.021	−0.025	0.058
Direct effect	0.256	0.039	0.180	0.332
Total effect	0.273	0.037	0.201	0.345

Note: AHWC: after-hours work connectivity; APJC: approach-oriented job crafting; AVJC: avoidance-oriented job crafting; JP: job performance.

**Table 6:** Results of the moderating effects test.

Variables	Approach-Oriented Job Crafting			Avoidance-Oriented Job Crafting		
	Model 1	Model 2	Model 3	Model 4	Model 5	Model 6
Control variables						
Gender	0.039	0.020	0.030	0.013	0.041	0.029
Age	−0.057	−0.073	−0.061	−0.058	−0.048	−0.061
Education level	0.068	0.093 *	0.086	−0.067	−0.054	−0.047
Length of service	0.051	0.017	0.022	0.056	0.075	0.069
Job rank	0.170 **	0.099 *	0.076	−0.042	−0.04	−0.014
Independent variable						
AHWC		0.231 ***	0.234 ***		0.236 ***	0.233 ***
Moderator variable						
PC		0.355 ***	0.349 ***		−0.293 ***	−0.287 ***
Interaction term						
AHWC * PC			0.142 **			−0.155 **
R2	0.035	0.260 ***	0.280 **	0.010	0.102	0.125
∆R2		0.226	0.019		0.092	0.023
F	2.879 *	20.051 ***	19.308 ***	0.772	6.471 ***	7.114 ***
∆F		60.831 ***	10.696 **		20.530 **	10.534 **

Note: AHWC: after-hours work connectivity; PC: psychological contract. *** *p* < 0.001, ** *p* < 0.01, * *p* < 0.05.

**Table 7:** Mediating effects at different levels of the psychological contract.

Mediating Variable	Mediator			Index of Moderated Mediation	
	Moderator	Effect	(CI)	Index	(CI)
Approach-Oriented Job Crafting	Low Moderation	0.012	[−0.008, 0.034]	0.028	[0.007, 0.058]
	High Moderation	0.050	[0.018, 0.087]		
	Between-Group Difference (High-Low)	0.038	[0.009, 0.079]		
Avoidance-Oriented Job Crafting	Low Moderation	−0.082	[−0.127, −0.044]	0.049	[0.018, 0.086]
	High Moderation	−0.016	[−0.045, 0.012]		
	Between-Group Difference (High-Low)	0.066	[0.024, 0.117]		

## Data Availability

The raw data supporting the conclusions of this article will be made available by the authors on request.

## Appendix A

The following are the survey items.

After-Hours Work Connectivity:

1. During non-work time (before work, during lunch breaks, after work, and on weekends and holidays), individuals related to work contact me via phone calls, emails, or messages (such as DingTalk, WeChat, Feishu).
2. During non-work time, how often I use communication tools to contact work-related people for work-related matters.
3. During non-work time, how often I check for work-related messages during non-work time.

Approach-Oriented Job Crafting:

1. I express my opinions on important issues and provide suggestions for the team or organization.
2. I increase the variety of my work activities and invest more time and effort in work that I enjoy and that is meaningful to me.
3. I make some extra effor to obtain resources (materials, information, support) that will help me in my work.
4. I do extra work (review changes, seek feedback) to ensure that the work I deliver is of higher quality.
5. I introduce new work activities or measures (safety inspections, safety equipment) to enhance the safety of the work environment.
6. At work, I proactively interact with others.
7. I work to improve my communication skills with others.
8. At work, I actively expand my professional network.
9. At work, I try to improve the quality of team interaction.
10. I build an orderly workflow to ensure that tasks are carried out in an organized manner.
11. I create a well-organized work environment (organize desk, manage documents).
12. I create a work schedule to organize my work.
13. I develop a work plan and prioritize my work in an organized manner.
14. I use new knowledge or technology to improve communication.
15. I use new knowledge or technology to automate tasks.
16. I independently seek and participate in training in new technologies.
17. I participate in self-directed training to improve my work.
18. I try to use new knowledge or technology to simplify my work.

Avoidance-Oriented Job Crafting:

1. I tend to do my work independently and avoid others’ involvement.
2. I would like to try not to communicate with others at work.
3. I would like to avoid trouble as much as possible in my work.
4. I try to get others to attend meetings instead of me.
5. I find ways to transfer my work to people outside my team.
6. I think of ways to reduce the time spent in the meeting.
7. I find ways to avoid time-consuming tasks.

Job Performance:

1. I improve my work efficiency by enhancing my overall quality.
2. I like to seek challenging work.
3. My work quality is high and I don’t often make mistakes.
4. I am willing to do my best to do a good job.
5. I am consistently able to complete tasks within the allotted time.
6. I am able to achieve the planned work objectives.
7. According to the current work situation, I will work efficiently through innovation.
8. I often take the initiative to take on jobs other than my own.
9. My work results are recognized by people around me.
10. I fulfill my work tasks in accordance with my own expectations and the requirements of my superiors.
11. I am very concerned about the company’s situation, in order to the development of the company I am willing to make efforts.
12. I take the initiative to communicate with coworkers and maintain good relations.

Psychological Contract:

1. The organization provides me with a safe and comfortable working environment.
2. The organization provides me with a stable income security.
3. The organization gives me a salary that matches my efforts.
4. The organization provides me with reasonable welfare benefits.
5. Mutual respect with my superiors and colleagues at work.
6. Acknowledgement of my work performance ait work.
7. My work can be recognized by my superiors and leaders.
8. The organization cares about my growth and life.
9. The organization provides me with better learning and training opportunities.
10. The organization has a perfect promotion system.
11. My work is challenging.
12. The organization gives me a certain degree of autonomy in my work and provides help when needed.
13. I have the right to participate in decision-making at work.

Demographic Information:

1. Gender: (Male/Female)
2. Age: (25 and below/26–35/36–45/46 and above)
3. Educational Background: (High school (junior college) degree or less/College degree/Bachelor’s degree/Master’s degree or above)
4. Years of Service in the Organization: (1 year and below/1–5 years/6–10 years/10 years and above)
5. Job Levels: (Ordinary employee/Front-line manager/middle manager/senior executive)
